# Synaptojanin 1 Mutation in Parkinson's Disease Brings Further Insight into the Neuropathological Mechanisms

**DOI:** 10.1155/2014/289728

**Published:** 2014-09-16

**Authors:** Valérie Drouet, Suzanne Lesage

**Affiliations:** ^1^Sorbonne Universités, UPMC (Paris 6), UMR S 1127, Inserm U 1127, CNRS UMR 7225, and ICM, 75013 Paris, France; ^2^Hôpital Pitié-Salpêtrière, Institut du Cerveau et de la Moelle Epinière ICM, 4ème Étage, 47 Boulevard de l'Hôpital, 75651 Paris, France

## Abstract

Synaptojanin 1 (SYNJ1) is a phosphoinositide phosphatase highly expressed in nerve terminals. Its two phosphatase domains dephosphorylate phosphoinositides present in membranes, while its proline-rich domain directs protein-protein interactions with synaptic components, leading to efficient recycling of synaptic vesicles in neurons. Triplication of SYNJ1 in Down's syndrome is responsible for higher level of phosphoinositides, enlarged endosomes, and learning deficits. SYNJ1 downregulation in Alzheimer's disease models is protective towards amyloid-beta peptide (A*β*) toxicity. One missense mutation in one of SYNJ1 functional domains was recently incriminated in an autosomal recessive form of early-onset Parkinson's disease (PD). In the third decade of life, these patients develop progressive Parkinsonism with bradykinesia, dystonia, and variable atypical symptoms such as cognitive decline, seizures, and eyelid apraxia. The identification of this new gene, together with the fact that most of the known PD proteins play a role in synaptic vesicle recycling and lipid metabolism, points out that synaptic maintenance is a key player in PD pathological mechanisms. Studying PD genes as a network regulating synaptic activity could bring insight into understanding the neuropathological processes of PD and help identify new genes at fault in this devastating disorder.

## 1. Introduction

Synaptojanin 1 (SYNJ1) was discovered in 1994 as a 145 kDa protein that interacts with growth factor receptor-bound protein 2 (Grb2) and a phosphoprotein involved in synaptic vesicle endocytosis and recycling [1, 2]. One year ago, a SYNJ1 mutation was incriminated for the first time in autosomal recessive early-onset Parkinson's disease (PD) with atypical symptoms [3, 4]. During this time, numerous studies were conducted on SYNJ1 and helped us better understand the roles of this multifunctional protein. This review will discuss how SYNJ1 operates in pre- and postsynaptic compartments to modulate synaptic activity, as well as its implication in different neurological disorders. SYNJ1 involvement in PD is also examined within the network of other known PD proteins.

## 2. SYNJ1 Gene and Protein Organization

**Figure 1:**
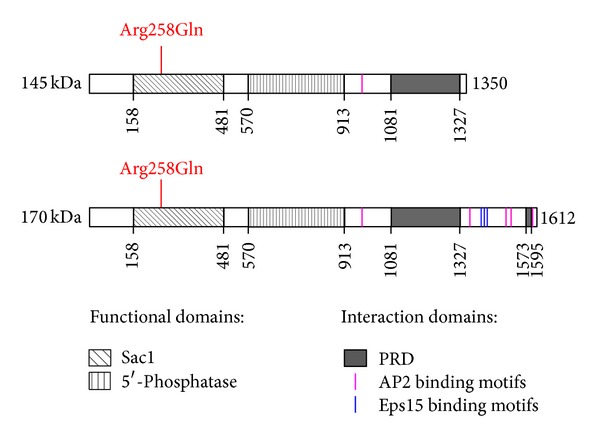
Functional and interaction domains of the two major isoforms of SYNJ1. The 145 kDa (top) and the 170 kDa (bottom) SYNJ1 isoforms harbor two functional inositol phosphatase domains, an N-terminal Sac1 domain and a more central 5′-phosphatase domain. Several protein-protein interaction domains are found in the C-terminal part of the proteins: one or two PRD domains, AP2 binding motifs (WxxF, FxDxF, and DxF, in pink), and Eps15 binding motifs (NPF: asparagine-proline-phenylalanine, in blue). The homozygous mutation Arg258Gln, found in Parkinson's disease patients, is indicated in red. Numbers indicate the amino acid positions along the proteins. Sac1: suppressor of actin1; PRD: proline-rich domain; AP2: adaptor protein complex 2; Eps15: epidermal growth factor receptor pathway substrate 15.

The SYNJ1 gene is located on chromosome 21q22.11 [5] and spans 99.29 kb of genomic DNA. Two isoforms of 170 and 145 kDa have been widely studied [2, 6, 7] (isoform a: NP_003886.3, 1612 amino acids; and isoform b: NP_982271.2, 1350 amino acids). They are generated from two open reading frames (ORFs) separated by an in-frame TAA stop codon [8]. They are both ubiquitously expressed, but the 145 kDa isoform is found in very high concentrations in brain [7, 8] where it is localized on coated endocytic intermediates in nerve terminals [2, 9]. Both isoforms harbor multiple functional domains: a suppressor of actin1 Sac1-like domain on their N-terminal, a 5′-phosphatase domain in the center, and a C-terminal proline-rich domain (PRD). The longer 170 kDa isoform contains an additional PRD translated from the second ORF (Figure 1). There are two additional SYNJ1 isoforms listed in RefSeq (isoform c: NP_001153774.1, 1295 amino acids; isoform d: NP_001153778.1, 1526 amino acids) that are of unknown functional relevance. Despite isoforms c and d have a shorter N-terminus and a distinct C-terminus and are shorter than isoform a, they contain the same functional domains as isoforms b and a, respectively.

### 2.1. Inositol Phosphatase Functions

Inositol lipids are essential components of eukaryotic membranes and important intracellular second messengers that can be regulated by phosphorylation. Inositol phosphatases remove phosphate groups from phosphoinositides (e.g., phosphorylated inositol lipids) and play important roles in lipid signaling, cell signaling, and membrane trafficking [10]. SYNJ1 contains two consecutive inositol phosphatase domains, the Sac1 and the 5′-phosphatase domains (Figure 1). The N-terminal Sac1 domain, homologous to the yeast SacIp, dephosphorylates predominantly phosphatidylinositol monophosphates present in cell membranes, including those of the Golgi apparatus and endosomes, to recruit proteins. The central 5′-phosphatase domain dephosphorylates phosphatidylinositol bis- or trisphosphates, localized in plasma membranes, to activate downstream pathways [6, 8, 11, 12]. In neurons, SYNJ1 dual phosphatase activity is required for efficient synaptic vesicle endocytosis and reavailability at nerve terminals [13].

### 2.2. Protein-Protein Interactions

Many intracellular proteins contain proline-rich sequences that serve as binding sites for Src homology 3 (SH3) domains. Based on its SH3 binding ability, SYNJ1 was initially identified as interacting with Grb2 [1]. In fact, SYNJ1 contains a 250 amino acid PRD at its C-terminus, with at least five potential SH3 domain-binding consensus sequences [8, 14]. The 170 kDa isoform harbors an additional smaller PRD with at least three additional SH3 binding sites [7] (Figure 1).

Besides Grb2, the C-terminal region common to both SYNJ1 isoforms interacts with the SH3 domains of a variety of proteins implicated in endocytosis, subcellular targeting, and signaling: endophilin, amphiphysin, syndapin/pacsin, intersectin, and many others [15–20]. Through a SH3-PRD interaction, endophilin recruits SYNJ1 to endocytic sites to promote dephosphorylation of phosphatidylinositol 4,5-bisphosphates by way of SYNJ1 5′-phosphatase activity [21]. The 170 kDa splice variant bears an additional C-terminal tail that contains binding sites for clathrin, clathrin adaptor protein complex 2 (AP2) via three types of binding motifs (WxxF, FxDxF, and DxF), and the epidermal growth factor receptor pathway substrate 15 (Eps15) through asparagine-proline-phenylalanine (NPF) domain [9, 22, 23] (Figure 1). The complex AP2 is a protein interaction hub binding to all the endocytic components, including Eps15, necessary for clathrin-mediated endocytosis [22].

## 3. SYNJ1 Multiple Functions

Because of its different functional domains, SYNJ1 plays a key role in nerve terminals, coupling endocytic vesicle fission, and phosphoinositide dephosphorylation, but it has also been shown that SYNJ1 takes part in similar mechanisms in cone photoreceptors [24–27], hair cells [28], podocyte foot processes [29], and, more recently and unexpectedly, T cells [30].

### 3.1. Functions in Neurons

SYNJ1 functions in neurons are mainly promoted by the 145 kDa isoform, since the 170 kDa isoform is undetectable in the adult rat brain [7]. Most of the studies focused on synapses, since SYNJ1 145 kDa is highly enriched in presynaptic nerve terminals and, like dynamin, interacts with amphiphysin and undergoes dephosphorylation after nerve terminal depolarization [2, 8]. It also interacts with endophilin and, together, they are rapidly recruited to clathrin-coated pits during prolonged stimulation [6, 13].

SYNJ1-deficient mice exhibit neurological defects such as severe weakness, ataxia, spontaneous epileptic seizures, and poor motor coordination and die shortly after birth [11]. Likewise, mutations in* unc-26*, the single synaptojanin gene in* C. elegans*, give rise to small animals which are moving backwards with a jerky motion and frequently coil and have reduced numbers of enteric muscle contractions [31]. Studies of these mutants, lamprey giant reticulospinal axons microinjected with antibodies against synaptojanin [32], and yeast inactivated for synaptojanin-like proteins [33] have revealed increased levels of phosphatidylinositol 4,5-bisphosphates, an increased number of clathrin-coated vesicles, and a hypertrophy of the actin-rich matrix at endocytic zones. This shows that, in the brain, regulation of phosphoinositide levels by the SYNJ1 5′-phosphatase domain is essential for proper vesicle trafficking and coating/uncoating of endocytic vesicles [11, 34]. Through dephosphorylation of phosphatidylinositol 3- and 4-monophosphate [12, 34], the SYNJ1 Sac1 domain participates in actin cytoskeleton polymerization/depolymerization and is mostly required during brief neuronal stimulation [13]. To a lower extent, Sac1 activity is also an arbiter of phosphatidylinositol 3,5-bisphosphates levels, playing an important role in early and late endosomes turnover [35]. In addition, another role has been identified for SYNJ1 postsynaptically: it is involved in the internalization of AMPA receptors in postsynaptic neurons [36].

Therefore, SYNJ1 not only is involved in endocytic and postendocytic mechanisms presynaptically but is also participating in the signal transmission through postsynaptic reorganization.

### 3.2. The Particular Case of Sensory Neurons

In the particular case of photoreceptor and hair cells, sensory information transmission relies on ribbon synapses. These “unconventional” synapses have very high rates of continuous exocytosis and therefore need to have efficient endocytosis and vesicle recycling mechanisms [37].

Mutation in SYNJ1 in a Zebrafish vision mutant (*nrc*) showed unanchored ribbons and reduced numbers and abnormal distribution of synaptic vesicles that are scattered within a dense cytoskeletal matrix in cone photoreceptors [25]. Additional studies in Zebrafish confirmed that SYNJ1 is required for proper membrane and protein trafficking at the ribbon synapses in cones [24, 26]. A mutation was also found in another Zebrafish with a vestibular deficit (*comet*) [28]. This model showed that SYNJ1 plays a critical role in facilitating vesicle recycling at ribbon synapses through controlling the number of vesicles released and timing of release. These roles discovered in Zebrafish have yet to be confirmed in mammalian models.

### 3.3. Functions in Other Cell Types

In the kidney, adjacent podocytes form an epithelial barrier via their foot processes, which are connected by a thin diaphragm (the slit diaphragm) for filtering plasma into the urinary space. In podocytes, only the 170 kDa isoform of SYNJ1 is expressed, and, like in neurons, SYNJ1 participates in endocytosis with its interacting partners dynamin and endophilin by acting on phosphoinositides and actin filaments [29]. This is required for an efficient glomerular filtration and thus for proper renal function.

Recently, SYNJ1 has been reported as a potential regulator of allogeneic T cell responses [30]. This phenomenon can be triggered after transplantation from a genetically different person. The level of SYNJ1 mRNA was reduced after allogeneic stimulation of naïve T cells [30]. The authors believe that this reduced expression level is due to specific miRNA targeting the SYNJ1 transcript. Knockdown of SYNJ1 in allogeneically stimulated T cells confirmed its role in T cells proliferation and cytokine responses [30].

## 4. SYNJ1 in Down's Syndrome (DS) and Alzheimer's Disease (AD)

The critical importance of SYNJ1 at synapses has led multiple teams to investigate its role in neurological disorders such as DS and AD. It became clear that a proper dosage of this gene was essential for good synaptic function.

### 4.1. SYNJ1 Trisomy in DS

DS, also known as trisomy 21, is the most common genetic cause of mental retardation and is caused by overexpression of one or several genes on chromosome 21. Along with the early development of AD pathology and muscle hypotonia, mental retardation occurs in all DS-affected individuals, whereas other phenotypes (e.g., congenital heart defects) occur in a fraction of patients [38]. Linkage analysis led to defining small chromosome 21 subregions as responsible for mental retardation, and SYNJ1 became a strong candidate gene [39]. Using a DS mouse model carrying a partial trisomy of mouse chromosome 16 (conserved with the long arm of human chromosome 21), it was shown that SYNJ1 was overexpressed in DS mouse brains, which was associated with higher levels of phosphatidylinositol 4,5-bisphosphates phosphatase activity and learning deficits [39]. Additional studies in human blood and* Drosophila* also confirmed the involvement of SYNJ1 in DS [40, 41]. In particular, its triplication triggers abnormal synaptic morphology in fly neuromuscular junctions [40] and enlargement of early endosomes in lymphoblastoid cell lines derived from DS patients [41]. These endosomal abnormalities have been implicated in the early development of AD pathology in DS patients, but amyloid precursor protein (APP, also triplicated in DS) overexpression alone is not responsible for inducing endosomal enlargement in DS lymphoblastoid cells [41]. Measurement of SYNJ1 protein levels in DS-affected brains showed higher levels compared to matched controls, which is in agreement with SYNJ1 triplication in DS [42]. Moreover, in brains from individual with DS + AD pathology, levels of SYNJ1 are even higher and correlate with levels of amyloid-beta peptide (A*β*), whereas SYNJ1 levels are reduced in sporadic AD brains. The authors suggest that higher A*β* level could reduce SYNJ1 turnover in DS + AD brains [42]. However, there are other genes that are triplicated in DS, and they could be involved, alone or together with SYNJ1, to explain the deficits observed in DS patients.

### 4.2. SYNJ1 in AD

DS patients, who carry triplication of both SYNJ1 and APP, develop early-onset AD [38]. This could be the result of overexpression of APP only, but some lines of evidence argue in favor of the combined effects of these two genes in the development of AD pathology. Both A*β* and SYNJ1 trigger internalization of AMPA receptor, which could provoke synaptic dysfunction [36, 43, 44]. In hippocampal cultures, addition of A*β* oligomers provoked a loss of dendritic AMPA receptors, via calcineurin-mediated endocytosis [44]. On the contrary, hippocampal neurons from SYNJ1 knockout mice showed more surface-exposed AMPA receptors [36]. Additionally, downregulation of phosphatidylinositol 4,5-bisphosphates enhances the production of A*β*42, while haploinsufficiency of SYNJ1 protects cells from the neurotoxic actions of A*β*42 [45]. The beneficial impact of SYNJ1 reduction in AD was confirmed in a mouse model of AD [46]. In these animals, hemizygous deletion of SYNJ1 rescued deficits in learning and memory. Moreover, genetic disruption of SYNJ1 attenuated A*β* oligomer-induced changes in dendritic spines of cultured hippocampal neurons [46]. This protective effect was shown as a result of a decrease in amyloid plaque burden mediated through accelerating endosomal/lysosomal degradation of A*β* [47]. These data underline the potential of SYNJ1 reduction as a possible therapeutic strategy to counteract AD pathology.

## 5. SYNJ1 Mutation in Parkinson's Disease (PD)

An abnormally high level of SYNJ1 is potentially responsible for several pathological features in DS, and reduction of this protein is being investigated as a therapeutic strategy to counteract AD. But what happens when this protein is mutated? Several studies have linked bipolar disorder (BPD) to chromosomal region 21q22 containing SYNJ1 in a subset of families. Additionally, genes coding for proteins involved in the regulation of synaptic vesicle function are potential candidates for the development of psychiatric disorders. Therefore, SYNJ1 was found as a good candidate for BPD. Nevertheless, after screening about 230 patients with BPD, Lachman's team failed to statistically implicate SYNJ1 in BPD [48, 49]. It was only last year that a mutation in SYNJ1 gene was associated, for the first time, to a neurodegenerative disorder, PD.

### 5.1. SYNJ1-Associated PD Mutation

In June 2013, using homozygosity mapping followed by exome sequencing, two teams independently identified the same homozygous mutation, Arg258Gln, in two consanguineous families, one Italian (from Sicilia) and one Iranian, suffering from autosomal recessive early-onset Parkinsonism [3, 4].

This missense Arg258Gln mutation that localizes in exon 5, within the Sac1 domain of the protein (Figure 1), is predicted to be damaging by multiple prediction programs, and the arginine in position 258 is conserved in thirteen SYNJ1 orthologs and five Sac1-like domains containing proteins [3, 4]. Additionally, this mutation impairs the Sac1 phosphatase activity targeting phosphatidylinositol monophosphate, suggesting that impaired synaptic vesicle recycling could be involved in PD pathology [3].

Screening of all exons in 138 additional patients, among which 46 presented with complex early-onset Parkinsonism, did not identify any other homozygous or compound heterozygous mutation in SYNJ1 [3, 4]. A team from Germany screened 792 PD patients (mostly Germans) and could not find any mutation in SYNJ1 exon 5 [50]. However, sequencing of the whole SYNJ1 coding sequence was missing. In addition, there were only 50 out of the 792 patients who had an age at onset <30 years, which was found to be an important feature in SYNJ1-associated PD cases (Table 1). Arg258Gln was also absent from 1268 control chromosomes (180 healthy controls from southern Italy [4], 96 controls from Iranian ancestry [3], 92 Caucasian neurologically normal individuals [3], and 266 controls from EPIPARK cohort [50, 51]) and absent from multiple public databases representing more than 13,000 chromosomes [3, 4].

Recently, a third family was identified with the same homozygous Arg258Gln mutation in two siblings [52]. This family, from Naples in Italy, was not consanguineous, and haplotype study showed that the mutation had arisen independently in the ancestors of the two Italian families [4, 52].

SYNJ1 was named PARK20 (Online Mendelian Inheritance in Man, OMIM, 615530), even though mutations in this gene are extremely rare so far. To date, six early-onset PD patients (from three families with two affected siblings each) are carrying the homozygous Arg258Gln mutation. Their parents are all heterozygous for this variant while unaffected siblings are homozygous carriers for the wild-type allele or heterozygous mutation carriers [3, 4, 52]. Screening of all SYNJ1 coding regions in additional early-onset PD is mandatory, and particular attention should be paid to potential copy number variations and mutations at the compound heterozygous state.

### 5.2. SYNJ1-Associated PD Phenotype

A phenotypic variability is observed in the three families presenting SYNJ1 mutation. Nevertheless, PARK20 families can be described as early-onset atypical Parkinsonism, with onset in the third decade of life, and severe progression in the first stages followed by stabilization in later stages [53]. Main clinical features combine tremor, dystonia, bradykinesia, and a poor response to levodopa treatment. Additional atypical signs such as seizures, cognitive impairment, developmental delay, and oculomotor disturbances are variable. Indeed, generalized seizures are seen in the Iranian siblings while only one of the Italian affected patients suffered of an episode of clonic seizures. Eyelid apraxia is seen in both Iranian and Sicilian families but is absent from the Neapolitan family. Important and mild cognitive decline are observed in the Sicilian and Neapolitan families, respectively, but not described in the Iranian siblings. Finally, only the Neapolitan siblings had mild delay in reaching the child developmental milestones [3, 4, 52]. Of importance, the six SYNJ1-mutated patients were examined at different stages of disease progression, did not always undergo the same tests, and were taking different treatments; it could account for some of the observed clinical differences.

The clinical features of these six patients are summarized in Table 1.

### 5.3. Synaptic Vesicle Recycling in PD

**Figure 2:**
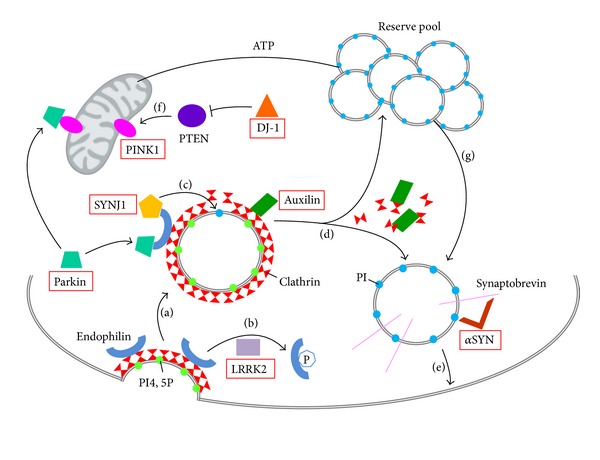
Synaptic vesicle recycling and PD genes. Schematic representation of a presynaptic terminal showing the PD genes (red boxes) and their role in synaptic vesicle recycling. (a) During endocytosis, invagination of the clathrin-coated membrane requires endophilin. Endophilin harbors several SH3 domains, which can interact with SYNJ1 PRD domain and/or parkin. (b) LRRK2 phosphorylates endophilin leading to dissociation of the later from clathrin-coated vesicles. (c) Once recruited to the coated vesicles through endophilin, SYNJ1 dephosphorylates PI4,5P into PI, shedding clathrin and its adaptor from the bilayer. (d) Uncoating of the vesicles also requires auxilin intervention and subsequent chaperoning of clathrin molecules. Then, the postendocytic vesicles can return to the reserve pool, where they undergo clustering, or return directly to the release site and enter in an exocytosis step. (e) Synaptic vesicles are docked and then fused to the membrane by means of a multiprotein complex including synaptobrevin and αSYN. (f) PTEN is a lipid phosphatase, which is inhibited by DJ-1, and can increase levels of the mitochondrial PINK1 protein. This pathway is involved in NMDA receptor signaling. (g) Proper mitochondrial functioning leads to ATP synthesis, necessary to mobilize the reserve pool of vesicles during synapse stimulation. PI4,5P: phosphatidylinositol 4,5-bisphosphates; PI: phosphatidylinositol; ATP: adenosine triphosphate; SYNJ1: synaptojanin 1; LRRK2: leucine-rich repeat serine/threonine-protein kinase 2; PTEN: phosphatase and tensin homologue; PINK1: PTEN induced putative kinase 1; DJ-1: Parkinson's disease protein 7; αSYN: alpha-synuclein.

The functions of SYNJ1 in synaptic vesicle recycling and actin dynamics in pre- and postsynaptic compartments are of high interest to understand the physiopathology of PD and, to a larger extent, the role of lipid metabolism in neurological disorders. There is mounting evidence that synaptic vesicle trafficking pathways are implicated in PD mechanisms. Most of the proteins involved in autosomal dominant PD, as well as those responsible for autosomal recessive forms of Parkinsonism, have been implicated, directly or indirectly, in synaptic vesicle turnover (Figure 2).

Parkin, an ubiquitin ligase mutated in the most common form of early-onset autosomal recessive PD, interacts with endophilin, which is a major binding partner of SYNJ1 (Figure 2(a)). Parkin participates in the ubiquitination of proteins present in synaptic endophilin complexes [54]. Leucine-rich repeat serine/threonine-protein kinase 2 (LRRK2), which is mutated in the most common form of autosomal dominant PD, regulates endophilin association to clathrin-coated vesicles through phosphorylation [55] (Figure 2(b)). Auxilin-1, which was recently identified in atypical early-onset PD, is also a direct partner of SYNJ1 during the process of uncoating synaptic vesicles [56]. SYNJ1 and auxilin-1 mutated patients show common features of early-onset Parkinsonism and seizures with other atypical symptoms. Furthermore, KO mice for each one of these genes show nearly identical phenotypes of defective synaptic vesicle recycling and severe neurological phenotype [11, 57]. Nevertheless, their roles are different in the mechanism: auxilin-1 is involved in clathrin disassembly and chaperoning, whereas SYNJ1 takes part in the adaptor shedding from the bilayer [57] (Figures 2(c) and 2(d)). Moreover, alpha-synuclein (αSYN), a presynaptic protein found accumulated in Lewy bodies in typical late-onset PD, is also implicated in synaptic vesicle exocytosis and recycling [58, 59]. It has been shown that αSYN binds to phospholipids via its N-terminus and to synaptobrevin-2 via its C-terminal extremity on synaptic vesicles surface, to promote vesicle fusion [58] (Figure 2(e)). Lastly, PINK1 (PTEN-phosphatase and tensin homologue-induced kinase 1), whose mutation is responsible for typical early-onset autosomal recessive PD, is mostly described as a mitochondrial protein. However, it has also been shown that PINK1 deficiency affects synaptic function, as the reserve pool of synaptic vesicles is not mobilized during rapid stimulation in PINK1-deficient* Drosophila *[60]. Furthermore, the fact that PTEN (1) is a lipid phosphatase, like SYNJ1 [10, 35], (2) induces PINK1 activity, and (3) is inhibited by DJ-1 [61], another autosomal recessive associated PD protein, strongly suggests involvement of lipid metabolism in PD (Figure 2(f)).

This network of proteins associated with synaptic vesicle pathways and PD strongly supports that impaired synaptic activity, resulting from altered lipid metabolism, is a key mechanism underlining the pathology. More studies in this direction should be conducted.

Also, other proteins, which are involved in synaptic activity and interact with known PD proteins, should be considered as good candidate for PD. However, each new gene discovered as causative in PD is only incriminated in a decreasing number of families. Whole exome sequencing technology should help us find additional patients carrying these mutations, but it is most likely that we are heading towards the discovery of private PD genes, for example, one gene = one family. It is going to become harder and harder to find common mutated genes in PD and therefore the validation of such candidate genes will be difficult.

## 6. Conclusions

SYNJ1 is a phosphoinositide phosphatase protein, which is required for proper synaptic activity. After being investigated as a candidate gene in bipolar disorder, Down's syndrome, and Alzheimer's disease with varying success, SYNJ1 was identified as the causative gene in three families with early-onset atypical Parkinsonism. One single homozygous mutation has been reported so far. SYNJ1 and most of other PD proteins play a role in vesicle recycling and lipid metabolism at the synapse; thus the study of these pathways is of particular interest to dissect the neuropathological processes involved and to find potential therapeutic targets to counteract PD.

## Figures and Tables

**Table 1:** Clinical features in patients with SYNJ1 homozygous Arg258Gln mutation: Iranian family [3], Sicilian family [4], and Neapolitan family [52] modified from [52].

	Iranian family		Sicilian family		Neapolitan family	
ID code	I	II	NAPO-16	NAPO-17	NAPO-41	NAPO-42
Gender	M	F	M	F	M	F
Consanguinity	Yes	Yes	Yes	Yes	No	No
Child developmental milestones	Not available	Normal	Normal	Normal	Delayed	Delayed
Seizures (age at onset)	Yes (3)	Yes (infancy)	No	No	One episode (uncertain)	One episode (16)
Age at PD onset	20	Early 20s	22	28	28	26
Symptoms at onset	Tremor, bradykinesia	Tremor, bradykinesia, eyelid twitching	Bradykinesia, fatigue, gait impairment, involuntary arm movements	Bradykinesia, speech and gait difficulties, involuntary arm movements	Bradykinesia	Tremor, bradykinesia
Age at last examination	29	39	50	34	31	27
Evolution	Eyelid apraxia and dysarthria at 22, generalized bradykinesia, limb rigidity, tremor, hypophonia, postural instability at 29	Similar to I + needed assistance to walk at 32, bedbound at 37, anarthric state, in fixed posture at 39	Cognitive decline, severe dysarthria, assistance needed at 23, anarthric state at 25, stooped posture, abnormal gait, axial and limb rigidity, impaired postural reflex, eyelid apraxia, mild dysphagia, dystonia, resting and action tremor at 47, stable at 50	Stooped posture, abnormal gait, impaired postural reflex, staring gaze at 31, resting and action tremor, axial and limb rigidity, dystonia, dysarthria, hypophonia, mild dysphagia, worsening dystonia and supranuclear gaze palsy at 34	Hypomimia, impaired speech, mild stooped posture, tremor, axial and limb rigidity, gaze limitation, dystonia, irritability, drooling and dysphagia at 31	Hypomimia, impaired speech, tremor and limb rigidity, slow gait, reduced postural reflex at 27
UPDRS-III score^$^ (age)	38 (29)	Not available	78 (47), 82 (50)	57 (31), 68 (34)	42 (31)	32 (27)
MMSE^$$^ (age)	Not available	Not available	Not administered due to severe motor and cognitive disability	26 (31), 24 (34)	28 (31)	24 (27)
Imaging data	Mild cortical atrophy, bilateral hyperintensity in white matter	Meningioma (surgically removed at 37)	Diffuse cortical atrophy, hyperintensity of hippocampi, thinning midbrain quadrigeminal plate, nigrostriatal dopaminergic deficit, cortical hypometabolism	Diffuse cortical atrophy, hyperintensity of hippocampi, thinning midbrain quadrigeminal plate, cortical hypometabolism	No gross abnormalities, nigrostriatal dopaminergic deficit, mild bilateral hypometabolism	No gross abnormalities, nigrostriatal dopaminergic deficit, mild bilateral hypometabolism
Response to levodopa	Not tolerated (severe dyskinesia)	Not tolerated (severe dyskinesia)	Not tolerated (dystonia, postural hypotension)	Not tolerated (dystonia, postural hypotension)	Not treated	Not treated

^$^UPDRS-III score: unified PD rating scale; higher scores indicate more severe Parkinsonism. Maximum score: 56 for Iranian family and 108 for Italian families.

^$$^MMSE: Minimental state examination; lower scores indicate lower cognitive performance. Maximum score: 30.
